# Investigating the utility of clinical outcome-guided mutual information network in network-based Cox regression

**DOI:** 10.1186/1752-0509-9-S1-S8

**Published:** 2015-01-21

**Authors:** Hyun-hwan Jeong, So Yeon Kim, Kyubum Wee, Kyung-Ah Sohn

**Affiliations:** 1 Department of Information and Computer Engineering, Ajou University, Suwon, S. Korea 443-749

## Abstract

**Background:**

Network-based approaches have recently gained considerable popularity in high- dimensional regression settings. For example, the Cox regression model is widely used in expression analysis to predict the survival of patients. However, as the number of genes becomes substantially larger than the number of samples, the traditional Cox or *L*_2_-regularized Cox models are still prone to noise and produce unreliable estimations of regression coefficients. A recent approach called the network-based Cox (Net-Cox) model attempts to resolve this issue by incorporating prior gene network information into the Cox regression. The Net-Cox model has shown to outperform the models that do not use this network information.

**Results:**

In this study, we demonstrate an alternative network construction method for the outcome-guided gene interaction network, and we investigate its utility in survival analysis using Net-Cox regression as compared with conventional networks, such as co-expression or static networks obtained from the existing knowledgebase. Our network edges consist of gene pairs that are significantly associated with the clinical outcome. We measure the strength of this association using mutual information between the gene pair and the clinical outcome. We applied this approach to ovarian cancer patients' data in The Cancer Genome Atlas (TCGA) and compared the predictive performance of the proposed approach with those that use other types of networks.

**Conclusions:**

We found that the alternative outcome-guided mutual information network further improved the prediction power of the network-based Cox regression. We expect that a modification of the network regularization term in the Net-Cox model could further improve its prediction power because the properties of our network edges are not optimally reflected in its current form.

## Background

The Cox regression model [1] has been commonly used in survival analysis to detect important features and to predict patient survival. Due to the advance of sequencing technology, the number of genes or features in these analyses is becoming substantially larger than the number of samples. Although several variant models, such as *L*_1 _−regularization [2] or *L*_2 _−regularization in Hilbert space [3], have been proposed to resolve this issue, those variant models are still prone to noise and over-fitting [4]. Recently, network-based approaches have gained considerable popularity for expression quantitative trait loci (eQTL) studies and clinical outcome predictions in high-dimensional regression settings. These approaches incorporate prior network information of either the features [5] or outcomes [6], or both [7,8]. For survival analysis, Zhang *et al*. [9] recently proposed a variant of the *L*_2 _−regularized Cox model called the Net-Cox model, which is a network-regularized Cox regression model. The typical types of networks used in such approaches are either coexpression networks, which are constructed by computing the correlation between every pair of variables, or static networks, such as the protein-protein interaction (PPI) network, which can be obtained from the existing knowledgebase. In the Net-Cox model, co-expression and functional linkage networks were incorporated in the survival analysis, and the results showed enhanced performance when compared with the conventional methods, which do not use the network information.

A potential limitation of these conventional networks is that the edges only reflect the information of within-features or within-outcomes relations, and do not consider the association between features and outcomes, which may be useful in improving the predictive power. In this study, we show that the outcome-guided mutual information network improves the performance of survival analysis in the Net-Cox regression [9]. We demonstrate the utility of this outcome-guided gene network with the analysis of a TCGA ovarian cancer dataset and compare its performance with those of survival analyses that use other types of networks.

## Methods

### Background on survival analysis using Cox regression

First, we describe the basic formulation of Cox regression [1] for survival analysis using expression and survival data. Given a gene expression profile *X*, which consists of *n *patients and *p *genes, the risk of an event at time *t *for the *i*th patient with gene expression X*_i _*= (X_i1 _,...,*X_ip_*)′ is defined as $h\left(t|X_{i}\right)=h_{0}\left(t\right)e^{{X^{\prime }}_{i}\beta }$, where *β *= (*β*_1_,...,*β_p_*)′ is a regression coefficient vector, and *h*_0_(*t*) denotes the baseline hazard function at *t*. The coefficient *β *and the function *h*_0 _are generally unknown and need to be estimated. In a traditional Cox regression model, the estimation of *β *is based on the maximization of the partial log-likelihood, *pl*(*β*):

$$pl\left(\beta \right)=\sum_{i=1}^{n}\delta_{i}\left\{{X^{\prime }}_{i}\beta -\text{log}\left[\sum_{j\in R\left(t_{i}\right)}e^{{X^{\prime }}_{j}\beta }\right]\right\}.$$

Here, *δ_i _*is the observed status (*δ_i _*= 1 implies observed; *δ_i _*= 0 implies censored), *t_i _*represents the event time of the *i*th patient, and *R*(*t_i_*) is the subset of patients who survived to time *t_i_*. Once the coefficient vector $\hat{\beta }$ is obtained, a Breslow estimator can estimate the baseline hazard function *h*_0 _as follows:

$${\hat{h}}_{0}(t_{i})=\frac{1}{\sum_{j\in R(t_{i})}e^{X{'}_{j}\hat{\beta }}}.$$

### Regularized Cox regression in high-dimensional setting

When *p *≫ 1, the Cox regression model is prone to noise and tends to produce unreliable estimations of regression coefficients. Several solutions that shrink the coefficients have been proposed. A common solution is the *L*_2 _−Cox model, which uses the penalized total log-likelihood:

$$l_{pen}\left(\beta ,h_{0}\right)=\overset{n}{\underset{i=1}{\sum }}\left\{-e^{X^{\prime }j\beta }H_{0}\left(t_{i}\right)+\delta_{i}\left[log\left(h_{0}\left(t_{i}\right)\right)+{X^{\prime }}_{\beta }\right]\right\}-\frac{1}{2}\lambda \overset{p}{\underset{j=1}{\sum }}\beta_{j}^{2},$$

where $\lambda \sum_{j=1}^{p}\beta_{j}^{2}$ is the regularization term and *λ *controls the amount of shrinkage. Net-Cox regression is an extension of the *L*_2 _−Cox model and uses the following penalized log-likelihood:

$$l_{pen}\left(\beta ,h_{0}\right)=\overset{n}{\underset{i=1}{\sum }}\left\{-e^{X^{\prime }j\beta }H_{0}\left(t_{i}\right)+\delta_{i}\left[log\left(h_{0}\left(t_{i}\right)\right)+{X^{\prime }}_{\beta }\right]\right\}-\frac{1}{2}\lambda \beta^{\prime }\Gamma \beta ,$$

in which *λβ*′Γ*β *is the penalty term and Γ = (1 - α) *L *+ *αI *conveys prior network information between genes. Here, *L *= *I *− *S*, where *I *is the identity matrix and *S *is a normalized Laplacian matrix, which comes from the gene network.

The parameter α ∈ (0,1] controls the contribution of network information to the model. When α is small, more network information is incorporated into the penalty term than when α is large. We note that when α = 1, the network-based cox regression model reduces to the *L*_2 _−Cox model that does not use any network information.

The typical network used for this type of network-based approach is the coexpression network, in which the edge weights correspond to the correlation between expression vectors of two genes [6]. In [9], a functional linkage network was constructed from the existing knowledgebase. We note that both types of networks do not reflect information about the relation between the features and the outcomes inferred from a given dataset. Therefore, we consider an alternative approach of constructing and utilizing outcome-guided mutual information network in survival analysis. The detailed procedures are explained in the following sections.

### Gene interaction networks associated with clinical outcome

Mutual information is an information-theoretic measure that reveals the dependency or association between two random variables [10] and is defined as follows:

I(X;Y)=H(X)+H(Y)-H(X,Y),

where *H*(*X*) and *H*(*Y*) denote the entropies of the respective variables, *X *and *Y*, and *H*(*X*, *Y*) denotes the joint entropy of *X *and *Y*. It can be used to detect both linear and nonlinear relations between two random variables [11]. While many previous studies generally used mutual information to detect dependency between two features of the same type, more recent approaches in [12,13] have also extended the mutual information to assess the association between expressions of two genomic features, *X*_1 _and *X*_2_, and the clinical outcome *Y *as follows:

$$I\left(X_{1},X_{2};Y\right)=H\left(X_{1},X_{2}\right)+H\left(Y\right)-H\left(X_{1},X_{2},Y\right).$$

Note that genomic feature values, such as gene expressions or clinical outcome values, are often numeric. To compute the mutual information, we discretize the genomic feature values using a histogram-based technique as in [14]. In the case of survival data, a simple thresholding scheme converts the outcome variable of the survival month to a binary variable. In the case of the TCGA dataset used in this study, we split the patients into short-term living (≤ 3 years) and long-term living (> 3 years). Patients who are reported as *living *and have an overall survival time less than 3 years are filtered out of this study.

Computation of mutual information for every pair of genes and the clinical outcome variable produce a complete network between genes. We can further filter out less significant edges by using the permutation testing scheme proposed in [14]. Through the repeated permutation of clinical outcome labels and the re-computation of mutual information values, a threshold θ is defined as the maximum of the average mutual information values for each gene pair. This θ can be used as a base threshold to remove insignificant edges. However, this threshold still leaves a large number of edges that may not be fully beneficial to the downstream analysis. In this study, another parameter *σ*, which amplifies the significance level by a factor of (1 + *σ*), was proposed to create a stricter cut-off. In particular, the filtered mutual information network with significance level *σ *is defined by

$${\text{G}}_{\sigma }=\left\{\left(g_{i},g_{j}\right)|g_{i},g_{j}\in P\;and\;I\left(g_{i},g_{j};Y\right)\geq \theta \left(1+\sigma \right)\right\},$$

where *P *is the set of all genes, *Y *denotes the binary survival status of patients, and *I*(*g_i_*, *g_j_*; *Y*) is the mutual information of the gene pair (*g_i_*, *g_j _*) and *Y*. We adopt this thresholding scheme and test the performance of different parameter values. In order to apply the network to the Net-Cox model, we also normalize G_*σ *_and create a normalized Laplacian matrix $\text{S}={\text{R}}^{-\frac{1}{2}}G_{\sigma }C^{-\frac{1}{2}}$, where ${\text{R}}_{ii}=\sum_{j}G_{\sigma_{ij}}$ and ${\text{C}}_{ii}=\sum_{j}G_{\sigma_{ij}}^{\prime }$ both of which are diagonal matrices.

### Dataset

We use genomic profiles and clinical outcome data from patients with *ovarian serous cystadenocarcinoma *in TCGA. Genomic profiles of copy number alterations (CNAs), messenger RNA (mRNA), and methylation (METH) are used in the experiments. We remove genes or patients with missing values and extract genes that are common to all three profiles. As a result, our data matrix consists of expressions and alterations of 10,022 genes and 340 patients for each of the three profiles.

### Evaluation measure

We compare the performance of the Net-Cox model using the proposed mutual information networks with those of models using a gene co-expression and gene functional linkage network as seen in [9]. In order to create a baseline, an analysis of the *L*_2 _− Cox model, which does not use any network information, is performed. This is the same as setting α = 1 in the Net-Cox model.

With each run of the Net-Cox analysis, the prognostic indices $PI=X^{\prime }\hat{\beta }$ are calculated as prediction markers, where *X *is the test data not used in training, and the regression coefficient $\hat{\beta }$ is estimated from given training data, network information, and the parameters. We evaluate the prediction performance using a time-dependent area under the curve (AUC) [15].

Specifically, time-dependent sensitivity and specificity functions are defined as follows:

$$\begin{array}{c} sensitivity\left[c,t|f\left(X\right)\right]=Pr\left\{f\left(X\right)>c|\delta \left(t\right)=1\right\},\\ specificity\left[c,t|f\left(X\right)\right]=Pr\left\{f\left(X\right)>c|\delta \left(t\right)=0\right\}, \end{array}$$

in which *c *is the cut-off point, *t *is the survival time, *f*(*X*) are the prognostic indices, $f\left(X\right)={\text{X}}^{\prime }\beta$and *δ*(*t*) is the event indicator at time *t *[16]. Upon examination of *sensitivity*[*c*, *t*|*f*(*X*)] and 1 - *specificity*[*c*, *t*|*f*(*X*)], we can define ROC[*t*|*f*(*X*)] as receiving operating characteristic (ROC) curves at any time *t *and AUC[*t*|*f *(*X*) ] as the area under the ROC curves at any time *t*. The larger AUC[*t*|*f*(*X*)] is, the better our prediction model performs at time *t*.

In addition, we evaluate the performance, using a validation set and the log-rank test [17], to analyze whether the patients are properly classified: high-risk or low-risk. We rank patients in descending order by their prognostic indices (*PI) *and divide them into a high-risk group containing the top 40% of patients and a low-risk group containing the bottom 40%. We then test the validity of this group assignment by using the log-rank test and the true survival information.

### Cross validation

We select the optimal parameters, λ and α, that respectively control the degree of sparsity and the amount of network constraint in the Net-Cox model using a 5-fold cross validation process. We reserve 20% of all the samples in this study for validation (the validation set) and use the remaining 80% (the training set) for cross validation in order to optimize the parameters and check the cross-validation error. In the case of mutual information networks, we also choose the optimal *σ *using cross-validation. The average time-dependent AUC is used as an evaluation measure for each genomic profile and the different kinds of networks.

We trained each model five times using a 5-fold cross validation process on the training set. At each run, we estimate the regression coefficient $\hat{\beta }$ and the mutual information network (or co-expression network) on four folds of the training data. The estimated coefficient $\hat{\beta }$ and the remaining fold, consisting of 20% of the training data, are then used to calculate the prognostic indices *PI *= *X*′*β*, which are, in turn, used to rank the patients according to their expected survival time, or month.

To examine how the mutual information network filtered by significance level *σ *contributes to the performance of the Net-Cox model, we experiment with the complete mutual information network and those networks filtered by *σ*, where *σ *= [0, 0.05, 0.1, 0.15, 0.2, 0.25, 0.3] for each profile. This corresponds to eight mutual information networks on each profile, including the complete network. We vary the parameters λ = [10^−4^,10^−3^, 10^−2^, 10^−1^] and α = [0.1, 0.3, 0.5, 0.7, 0.9, 1]. As a result, we choose the optimal (λ, α) pair with the best performance, in terms of the time-dependent ROC curves, for each network and each profile.

### Enrichment analysis

In order to assess the biological significance of the results, we performed a gene list enrichment analysis for the 100 genes with the largest regression coefficients for each profile using *ToppGene *http://toppgene.cchmc.org[18]. The enrichment analysis is based on Gene Ontology [19] and Pathway and Disease [20]. The terms that have adjusted *p*-values under 0.05 when using the Benjamini-Hochberg correction [21] are considered biologically significant. We also ran the enrichment test on the genes in the network consisting of the 100 edges with the largest mutual information values for each profile.

## Results and discussion

### Optimal parameter selection using cross validation

**Figure 1:**
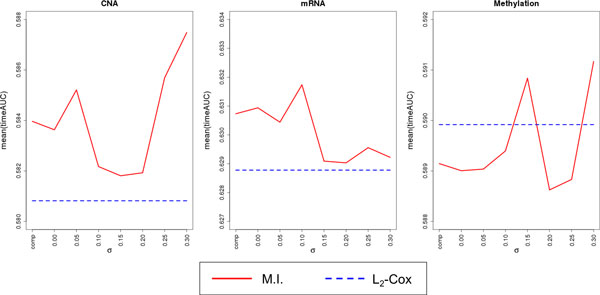
**Prediction accuracy of the mutual information network-based Net-Cox model as a function of the network significance parameter σ**. Average time-dependent AUC using 5-fold cross validation on training data is shown across σ.

We first examined the performance behavior of the mutual information network based Net-Cox model as a function of the parameter *σ *(Figure 1). Note that as the value of *σ *increases, fewer edges remain in the network. In Figure 1, the leftmost *x*-tick label "comp" represents the result obtained using the complete network, and the following *x*-ticks correspond to different *σ *values. Overall, the best performance resulted not from the complete network but from the network filtered by *σ*. Therefore, reducing less significant information and using only significant edges seem to improve the performance of the network-regularized regression model. When the CNA and methylation profiles were examined, the best result was obtained using the largest test value of *σ *= 0.3, for which 9,896 and 6,011 edges, respectively, remained, which was about 0.01% of the total number of pairs. In contrast, the mRNA profile preferred a larger network consisting of 568,486 edges (about 1%) with the optimum at *σ *= 0.1.

**Figure 2:**
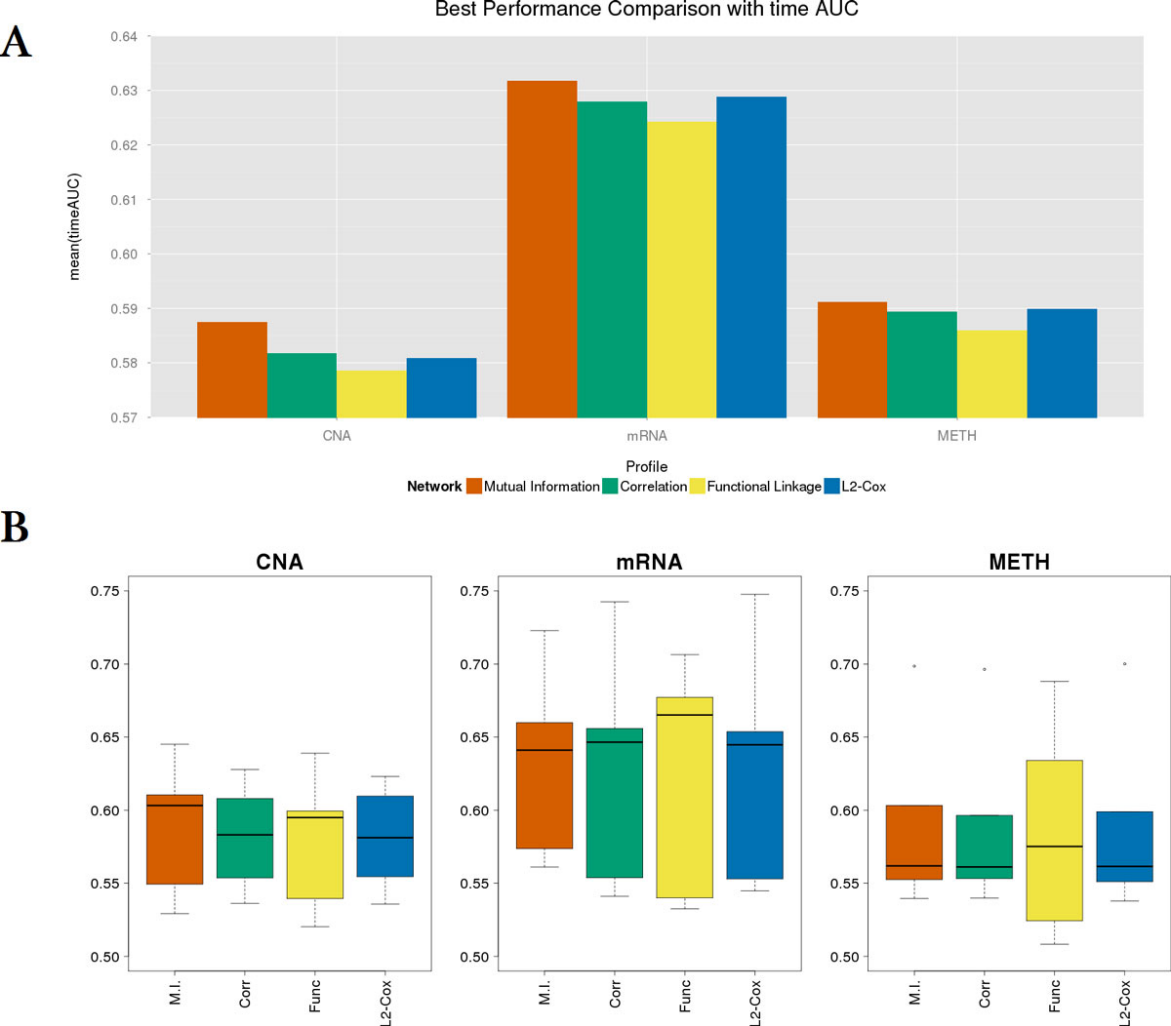
**Performance comparison of different network types in terms of 5-fold cross-validation accuracy**.

We also optimized the parameters *λ *and *α *using a 5-fold cross validation process (Table 1). We found that the optimal *α *value for the mutual information network was smaller than those of the co-expression and functional linkage networks. This implies that the mutual information network contributed more to the regression model than the other approaches. Moreover, the average time-dependent AUC was highest when the mutual information based network was used in all profiles. However, we also note that the median time-dependent AUC was not always highest for the mutual information network, which may imply complementary properties of different types of networks. In Figure 2, the bar plot for the average time-dependent AUC and the boxplot for the distribution of the time-dependent AUC across all the time points and the 5-fold experiments are shown.

**Table 1:** Optimal parameters and averaged time-dependent AUCs of each network using 5-fold cross validation on training data

Profile	Network	*σ*	*λ*	*α*	Mean (time AUC)
**CNA**	Mutual Information	0.30	10^−3^	0.1	**0.5875**
	Correlation	-	10^−3^	0.9	0.5817
	Functional Linkage	-	10^−3^	0.3	0.5786
	*L*_2 _− Cox	-	10^−3^	1.0	0.5810

**mRNA**	Mutual Information	0.10	10^−4^	0.1	**0.6317**
	Correlation	-	10^−4^	0.9	0.6280
	Functional Linkage	-	10^−4^	0.3	0.6242
	*L*_2 _− Cox	-	10^−4^	1.0	0.6288

**METH**	Mutual Information	0.30	10^−4^	0.5	**0.5912**
	Correlation	-	10^−4^	0.7	0.5894
	Functional Linkage	-	10^−4^	0.3	0.5860
	*L*_2 _− Cox	-	10^−4^	1.0	0.5899

### Performance comparison on validation set

**Figure 3:**
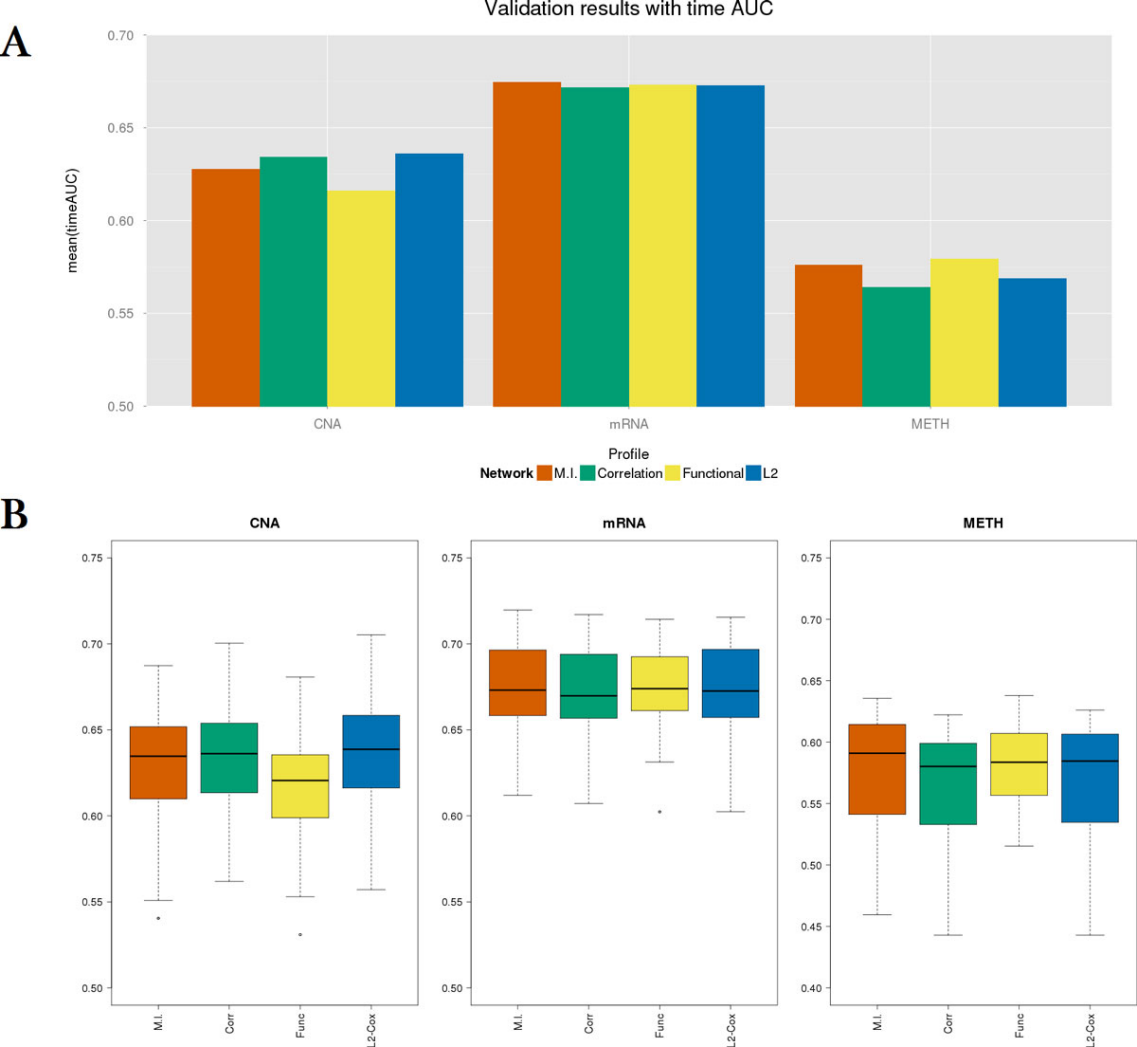
**Performance comparison of different network types on validation set**.

**Figure 4:**
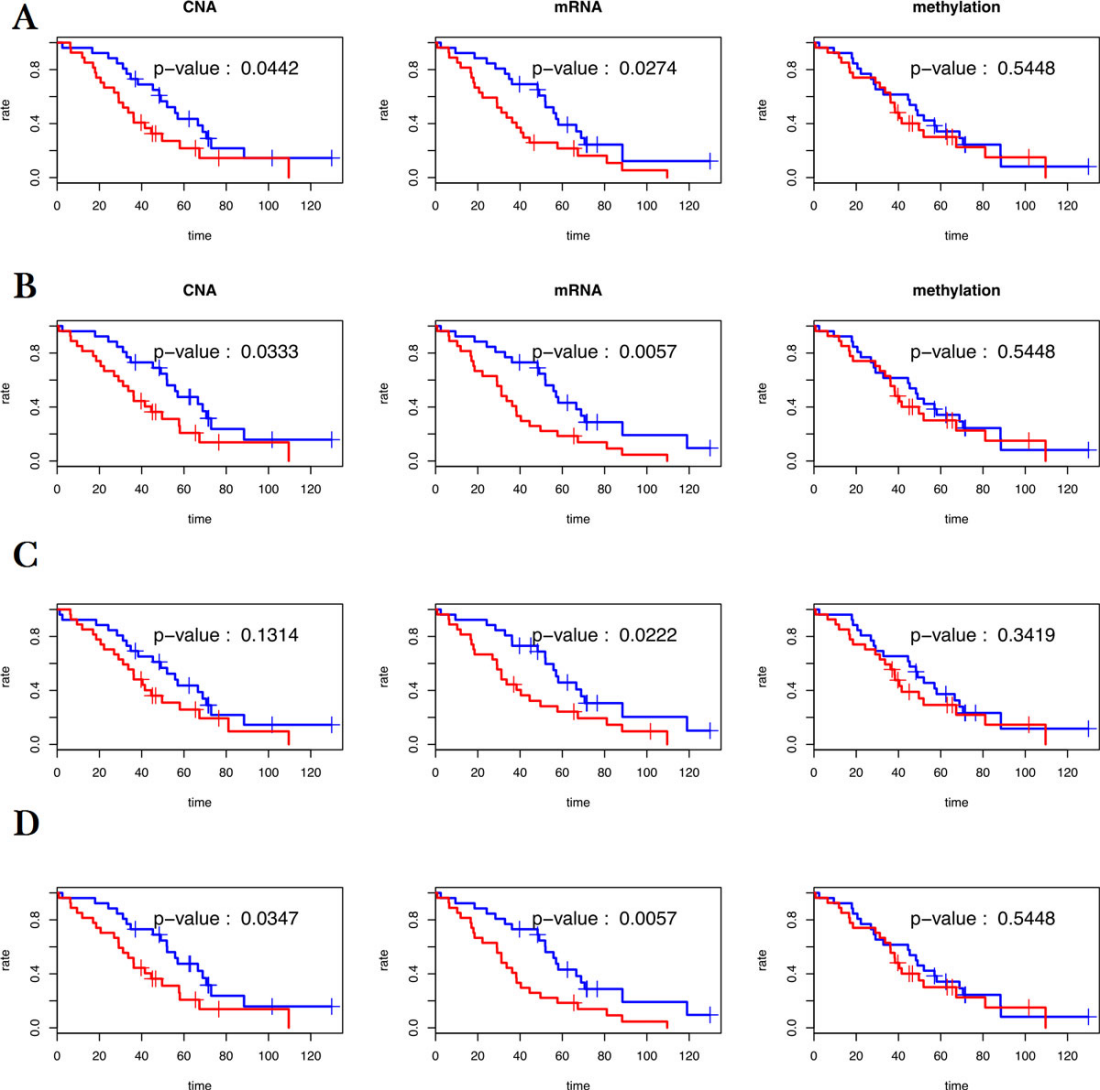
**Kaplan-Meier survival curves (high-risk group: red line; low risk group: blue line) and log-rank test results on validation set for each profile and network (A--Mutual information based network; B--Correlation network; C--Functional linkage network; D--*L*_2 _-Cox)**.

After selection of the best parameters for each network, we re-trained each model using the entire training set and then applied the obtained regression model to the holdout validation set. Figure 3 shows a comparison between the resulting time-dependent AUCs. The mutual information based network had the best performance for the mRNA profile and comparable results for the other profiles. Figure 4 shows the Kaplan-Meier survival curves and *p*-values from the log-rank tests with respect to the patient group assignment for each approach. Examining the CNA and mRNA profiles, every method revealed significant results (*p*-value < 0.05) with the log-rank test, except for the functional linkage network on the CNA profile. However, all the methods showed insignificant results for the methylation profile. It appears that the interaction effect in the methylation profile is substantially less than in other profiles.

### Signature genes for each profile

**Figure 5:**
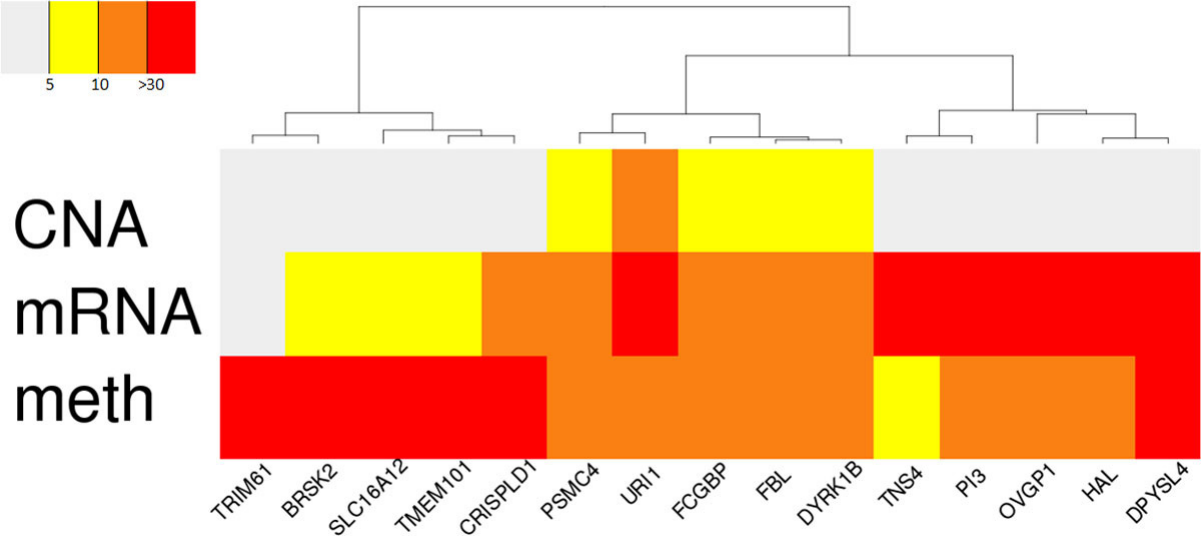
**Heat-map for the regression coefficients of 15 selected genes**. The five genes having the largest regression coefficients are selected for each profile and merged together.

In order to examine the genes that have the strongest marginal association with survival, we displayed the five largest regression coefficients in each profile as a heatmap (Figure 5). The top five genes from each profile were all distinct with no overlap, but some genes had large coefficients in multiple profiles. The top genes from the CNA profile had smaller overall regression coefficients than the top genes in other profiles. Moreover, the top genes in the CNA profile had larger regression coefficients than those in other profiles, which suggests that the roles of these genes are more prominent in other genomic levels.

We find that the identified genes are associated with ovarian cancer in many previous studies. The gene with the largest coefficient in the CNA profile was *URI1, prefoldin-like chaperone *(URI1), which is reported to be an oncogene that amplified in ovarian cancer cells [22]. *Dual-Specificity Tyrosine-(Y)-Phosphorylation Regulated Kinase 1B *(DYRK1B), which is over-expressed in a wide spectrum of ovarian cancer cell lines and human specimens [23], was the fourth highest gene. The largest coefficient gene from the mRNA profile, *Oviductal Glycoprotein 1 *(OVGP1), is reported to be a more accreted detection marker than other markers of ovarian epithelial cancers [24]. Over-expression of the *Elafin/Peptidase Inhibitor 3 *(PI3) gene from the mRNA profile is associated with poor overall survival [25]. We found no reported associations of the top five genes in the methylation profile with ovarian cancer from previous studies.

An enrichment analysis was performed for the 100 genes having the largest coefficients in each profile (Table 2). Many significantly enriched terms demonstrated an association with ovarian cancer. For example, the genes URI1, *Processing Of Precursor 4 *(POP4), *Pleckstrin Homology Domain Containing, Family F Member 1*(PLEKHF1), and DYRK1B from the CNA profile were enriched in the Ovarian Neoplasms term (ctd:D010051) of Disease. For Gene Ontology, the top 100 genes in the CNA profile were primarily enriched in the Biological Process (BP) terms. The Hox family of homeobox genes was enriched in the terms related with the embryonic skeletal system (GO:0048706; GO:0048704), and these genes are critical for cell migration and DNA repair [26]. The genes in the mRNA profile that were enriched in the Chitinases-related terms were already known for their cancer indication roles [27]. Therefore, we can conclude that the genes with large regression coefficients are expected to be related to ovarian cancer.

**Table 2:** Significantly enriched terms (using *ToppGene*) in the 100 genes that have the largest regression coefficients for each profile

Profile	Category	ID	Name	p-value	Adjusted p-value	Count	Total
**CNA**	GO:BP	GO:0048706	embryonic skeletal system development	5.99E-06	1.25E-02	7	129
	GO:BP	GO:0048704	embryonic skeletal system morphogenesis	1.43E-05	1.50E-02	6	98
	GO:BP	GO:0009952	anterior/posterior pattern specification	2.17E-05	1.52E-02	8	217
	Disease	ctd:D010051	Ovarian Neoplasms	5.24E-04	2.84E-02	4	78
	Disease	ctd:D002277	Carcinoma	7.42E-03	3.66E-02	4	161
	GO:BP	GO:0031442	positive regulation of mRNA 3'-end processing	7.94E-05	4.15E-02	3	16
	GO:BP	GO:0031440	regulation of mRNA 3'-end processing	1.15E-04	4.80E-02	3	18

**mRNA**	GO:MF	GO:0004568	chitinase activity	4.80E-06	1.78E-03	3	7
	GO:BP	GO:0006032	chitin catabolic process	4.83E-06	4.94E-03	3	7
	GO:BP	GO:0006030	chitin metabolic process	4.83E-06	4.94E-03	3	7
	GO:BP	GO:1901072	glucosamine-containing compound catabolic process	7.70E-06	5.24E-03	3	8
	GO:MF	GO:0086080	protein binding involved in heterotypic cell-cell adhesion	8.12E-05	1.50E-02	2	3
	GO:MF	GO:0098631	protein binding involved in cell adhesion	1.62E-04	1.50E-02	2	4
	GO:MF	GO:0098632	protein binding involved in cell-cell adhesion	1.62E-04	1.50E-02	2	4
	GO:BP	GO:0046348	amino sugar catabolic process	3.86E-05	1.97E-02	3	13
	GO:MF	GO:0008061	chitin binding	2.69E-04	1.99E-02	2	5
	GO:MF	GO:0030492	hemoglobin binding	7.45E-04	4.61E-02	2	8

**METH**	GO:CC	GO:0030424	axon	9.91E-06	1.95E-03	11	409
	GO:CC	GO:0043025	neuronal cell body	2.06E-04	1.57E-02	10	478
	GO:CC	GO:0043195	terminal bouton	3.15E-04	1.57E-02	4	62
	GO:CC	GO:0036477	somatodendritic compartment	3.19E-04	1.57E-02	12	705
	GO:CC	GO:0043005	neuron projection	4.07E-04	1.61E-02	14	945
	GO:CC	GO:0044297	cell body	5.10E-04	1.68E-02	10	536
	GO:CC	GO:0032983	kainate selective glutamate receptor complex	7.48E-04	2.11E-02	2	8
	GO:MF	GO:0004952	dopamine neurotransmitter receptor activity	2.80E-04	4.01E-02	2	5
	GO:MF	GO:0001965	G-protein alpha-subunit binding	3.95E-04	4.01E-02	3	27
	GO:MF	GO:0015075	ion transmembrane transporter activity	4.14E-04	4.01E-02	13	819
	GO:MF	GO:0022857	transmembrane transporter activity	5.21E-04	4.01E-02	14	951
	GO:MF	GO:0015026	coreceptor activity	5.98E-04	4.01E-02	3	31
	GO:MF	GO:0022891	substrate-specific transmembrane transporter activity	7.68E-04	4.29E-02	13	875
	GO:MF	GO:0022892	substrate-specific transporter activity	9.57E-04	4.58E-02	14	1012

### Network analysis

**Figure 6:**
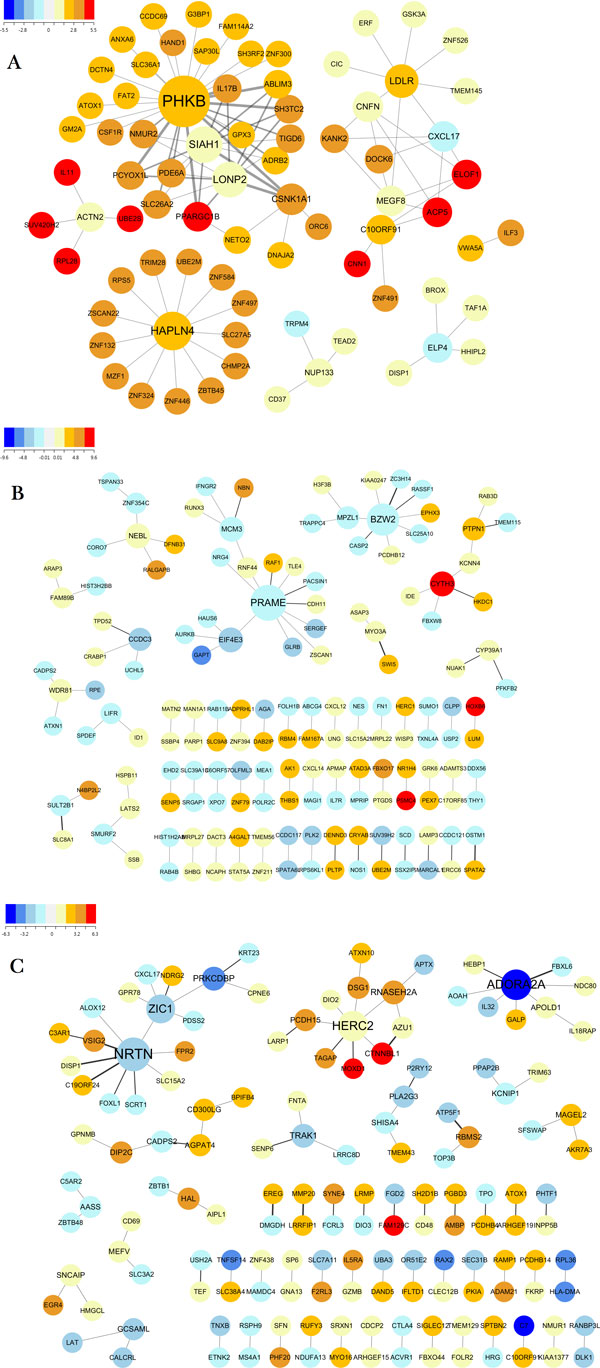
**Gene-gene interaction sub-network constructed by using the 100 edges with the largest mutual information values on each profile (A: CNA, B: mRNA, C: METH)**.

To illustrate the general topology of the mutual information network and its effect on prediction performances more closely, we constructed gene interaction sub-networks by using the 100 edges with the largest mutual information values for each profile (Figure 6). The color of each node represents the strength of its marginal effect. We also see many genes with weak marginal effects appear in the network. Topologies of the networks were analyzed by Cytoscape [28] and summarized in Table 3. The three networks reveal different network structures and topologies. The CNA network consists of a smaller number of connected components than the others (7 versus 54 and 50). It also shows a denser connection between the genes. The network centralization value is about five times higher, and its average number of neighbors is about twice as much. In addition, the overall coefficients of the genes in the CNA network were smaller than those in the other profiles. Considering that those genes have high mutual information values and, hence, strong interactive effects, this may imply that the interaction effect on survival in the CNA profile is more dominant than the marginal effect with each gene. The *R*^2 ^value for the power-law distribution of the mRNA and methylation networks were 0.922 and 0.909, respectively, which shows strong scale freeness [29], as is the case with many other biological networks [30-33].

**Table 3:** Network properties of mutual information sub-network for each profile

Properties	CNA	mRNA	METH
**Nodes**	78	154	149
**Connected components**	7	54	50
**Network centralization**	0.312	0.058	0.052
**Characteristic path length**	2.117	2.187	2.164
**Average number of neighbors**	2.564	1.299	1.342
**Network density**	0.033	0.008	0.009
**Network heterogeneity**	1.383	0.828	0.809
***R* ^2 ^** **of node degree distribution**	0.672	0.922	0.909

Table 4 summarizes the enrichment test results for the genes in each sub-network. The network from the methylation profile was mainly enriched in GO Molecular Function (MF) terms. Networks for the CNA and mRNA profiles were not enriched in GO terms but in terms of diseases related to ovarian cancer, such as Neoplasms (ctd:D009369), Carcinoma (ctd:D002277), and Neoplasm Recurrence, Local (ctd:D009364). Further, the CNA profile network was enriched in other related disease terms, such as Obesity (ctd:D009756) and BMI (Body Mass Index) [34], and Insulin Resistance (ctd:D007333) [35] and Hyperlipidaemia (ctd:D006949) [36].

**Table 4:** Significantly enriched terms (using ToppGene) in the sub-network consisting of the gene pairs that have the 100 largest mutual information values.

Profile	Category	ID	Name	p-value	Adjusted p-value	Count	Total
**CNA**	Disease	ctd:D006949	Hyperlipidemias	1.76E-05	2.24E-03	3	13
	Disease	ctd:D009765	Obesity	1.98E-03	1.21E-02	4	132
	Disease	ctd:D007333	Insulin Resistance	8.96E-03	2.42E-02	2	35
	Disease	ctd:D009369	Neoplasms	1.05E-02	2.78E-02	2	38

**mRNA**	Disease	ctd:D002277	Carcinoma	1.19E-04	1.23E-02	8	161
	Disease	ctd:D009364	Neoplasm Recurrence, Local	1.25E-02	4.96E-02	3	52

**METH**	GO:MF	GO:0008528	G-protein coupled peptide receptor activity	4.15E-05	1.21E-02	7	120
	GO:MF	GO:0001653	peptide receptor activity	4.86E-05	1.21E-02	7	123
	GO:MF	GO:0004942	anaphylatoxin receptor activity	1.78E-04	1.78E-02	2	3
	GO:MF	GO:0004948	calcitonin receptor activity	1.78E-04	1.78E-02	2	3
	GO:MF	GO:0004800	thyroxine 5'-deiodinase activity	1.78E-04	1.78E-02	2	3

## Conclusions

In this study, we investigated the utility of an alternative network construction approach based on mutual information in network-based Cox regression. Our results show that the mutual information based network can further improve prediction performance in survival analyses. Moreover, the permutation testing scheme used to discard insignificant pairs improved the prediction performance.

Overall, the performance gain of this alternative approach over existing methods was rather marginal. It seems due to a mismatch between the high mutual information value and the small value of the penalty term $\left(\psi \left(\beta \right)\right)=\frac{1}{2}\sum_{i,j}^{p}S_{i,j}{\left(\beta_{i}-\beta_{j}\right)}^{2})$ in the Net-Cox model--it does not necessarily mean that the gene pairs containing high mutual information with respect to survival should have similar marginal effects because the mutual information measure is more concerned with the interaction effect. Even with this discrepancy, results based on the mutual information network are still promising. In future studies, the regularization term could be modified to better reflect the information contained in the mutual information network and, hence, further improve the performance. Another direction would be to apply this network scheme to network-based approaches in other domains.

## Competing interests

The authors declare that they have no competing interests.

## Authors' contributions

HJ, KW, and KS designed the study. HJ and SK implemented the idea and performed the experiments. HJ, SK, KW, and KS developed the idea and performed the analysis. HJ, SK, KW, and KS wrote the paper.

## Declarations

The publication costs of this article were funded by Ajou University, S. Korea.

This article has been published as part of *BMC Systems Biology *Volume 9 Supplement 1, 2015: Selected articles from the Thirteenth Asia Pacific Bioinformatics Conference (APBC 2015): Systems Biology. The full contents of the supplement are available online at http://www.biomedcentral.com/bmcsystbiol/supplements/9/S1
